# Analyzing the effect of new technology and knowledge adoption on workers with visual impairment through a serial mediation model

**DOI:** 10.1038/s41598-026-40949-x

**Published:** 2026-06-29

**Authors:** Jing Bai, Pingping Fu, Wenjie Ye

**Affiliations:** 1 College of Pharmaceutical Business, Zhejiang Pharmaceutical University, 315500 Ningbo, China; 2 Nottingham University Business School China, University of Nottingham Ningbo China, 199 Taikang East Road, Ningbo, 315100 China; 3 The Department of Business Administration, Shanghai University of Finance and Economics Zhejiang College, Jinhua, 321000 Zhejiang China

**Keywords:** Adopting new technology and knowledge (ANTK), Work engagement, Person-job fit, Positive identity, JD-R model, Workers with visual impairments, Psychology, Human behaviour

## Abstract

Adopting new technology and knowledge (ANTK) has become a powerful force for enhancing productivity. This study examines the impact of ANTK on work engagement among visually impaired workers, with a focus on the mediating roles of person-job fit and positive identity as a visually impaired individual. Grounded in the Job Demands-Resources (JD-R) model, the study explores how ANTK serves as a critical job resource, mitigating the challenges associated with visual impairments and fostering higher levels of work engagement. Based on data from 204 visually impaired workers, the findings demonstrate that ANTK directly enhances work engagement. Moreover, person-job fit and positive identity sequentially mediate the relationship between ANTK and engagement. The findings provide theoretical contributions to both the JD-R model and the literature on disability and employment by highlighting the psychological mechanisms that link technology adoption to work outcomes. Practically, the study provides valuable insights for organizations seeking to leverage inclusive technologies to enhance accessibility, support identity development, and foster a more equitable and engaging workplace for workers with visual impairments.

**Supplementary Information:**

The online version contains supplementary material available at 10.1038/s41598-026-40949-x.

## Introduction

Adopting new technology and knowledge (ANTK) in the workplace has fundamentally transformed how workers interact with their tasks^1^. Increasingly, it is recognized that technologies can bridge gaps, create opportunities, enhance performance, and drive innovation in a rapidly evolving work environment^2,3^. However, most research on workplace technology adoption has focused on workers without disabilities, with limited empirical studies examining the effect of technology on work-related outcomes for workers with disabilities. Moreover, while technologies are often viewed as tools to reduce workplace barriers, few studies have examined how personal factors affecct the relationship between ANTK and work-related outcomes for this population.

For workers with disabilities, integrating assistive technologies is crucial for overcoming barriers and enhancing their participation in the workplace. According to the World Health Organization (WHO, 2024), approximately 1.3 billion individuals – or about 16% of the global population – are affected by disabilities. These staggering figures underscore the urgent need for both organizations and individuals to develop technology-driven solutions to reduce workplace barriers.

Among the diverse population of individuals with disabilities, those with visual impairments represent a significant subgroup. According to the International Agency for the Prevention of Blindness (IAPB, 2020), approximately 43 million people worldwide are affected by blindness, while 553 million individuals experience mild to severe vision impairments. These individuals often face barriers to information processing or accessibility, which can hinder their meaningful engagement in the workplace.

Recognizing the importance of “research on a specific disability type” to better understand career development or work-related behaviors^4^, this study aims to address the empirical gap by examining how the adoption of new technology and knowledge (ANTK) affects work engagement among workers with visual impairments. Specifically, the study explores the mediating roles of person-job fit (P-J fit) and positive identity as a person with a disability.

In the workplace, workers with visual impairments often encounter additional challenges. For instance, standard office software may not be compatible with screen readers, and inefficiencies may arise from inaccessible document formats, creating significant barriers that can reduce productivity and overall work engagement. An existing study provided evidence on how workplace design and access to technology impact work participation for individuals with visual impairments^5,6^. The findings show that these workers encounter invisible workload burdens—not only because of visual problems but because workplace devices are often not optimized for accessibility. Fortunately, the rapid evolution of technology has provided new opportunities to overcome these challenges. According to the unified theory of acceptance and use of technology, the four determinants (performance expectancy, effort expectancy, social influence, and facilitating conditions) affect new technology adoption, which in turn affects professional ability development and the capacity to participate fully in work processes^7^. By offering tools that make information accessible in alternative formats, assistive devices with new technologies can enable workers with visual impairments to complete tasks independently, improve efficiency, and foster greater participation in work activities^8,9^.

Despite the potential of these technologies, challenges persist. For example, the effectiveness of assistive technologies depends on how well they align with an individual’s skills, abilities, and job requirements – a concept known as person-job fit. Moreover, the adoption and effective use of assistive technologies are also affected by personal factors, such as a positive identity as a person with a disability. Workers who accept and embrace their disability are more likely to see technologies as empowering tools. In contrast, those with negative views of their disability may see these technologies as compensatory aids rather than as means of empowerment. Both external resources (i.e., technology) and internal personal resources (i.e., Person-Job fit and identity) play crucial roles in determining work engagement. Therefore, this study comprehensively examines how ANTK affects the engagement of workers with visual impairments, addressing both theoretical and practical concerns.

## Theoretical framework and hypotheses development

### The effect of adopting new technology and knowledge (ANTK) on work engagement

In the contemporary workplace, adopting new technology and knowledge (ANTK) has emerged as a key driver of productivity and engagement. For workers with disabilities, ANTK plays an essential role in overcoming barriers that would otherwise hinder their ability to engage in their work fully. For example, a prior study on individuals with intellectual and developmental disabilities in the workplace revealed that wireless or wearable technologies, when used as external resources, improve the working outcomes of this group^10^. ANTK in this study extends beyond the mere use of technological devices, but reflects the technology-related knowledge acquisition in work processes that enable employees to manage demands more effectively^11^. Specifically, ANTK encompasses two interrelated aspects: new technology adoption, which refers to the integration of new assistive and adaptive technologies, including screen readers, magnification software, braille displays, or other mobile assistive applications; and technology-related knowledge adoption, which involves developing and applying new skills, strategies, and cognitive resources to incorporate technology into daily work routines. It enables typical workers to align tasks with their capabilities, manage job demands, and pursue professional development, thereby reinforcing competence and engagement. Thus, we suppose that ANTK, a multidimensional job resource that integrates material and cognitive aspects of work adaptation, may positively affect work engagement among workers with visual impairment.

The Job Demands-Resources (JD-R) model posits that work-related outcomes, including job satisfaction, engagement, and productivity, depend on the balance between job demands and resources. Job resources encompass physical, psychological, cognitive, or social aspects that help employees achieve their work goals, reduce job demands, and foster personal development and growth^12,13^. In contrast, job demands refer to aspects that require emotional, cognitive, or physical effort, which can lead to strain and burnout if not adequately managed^14^. In our study, such a typical adoption can be conceptualized as an individual-level behavioral job resource. This behavioral conceptualization aligns with the JD–R model, in which job resources encompass aspects of the work environment that employees can proactively mobilize through action to mitigate job demands, achieve work goals, and foster learning and development. Accordingly, ANTK captures enacted behavioral efforts that enhance workers’ autonomy and accessibility of work information.

Work engagement is characterized by employees’ vigor, dedication, and absorption in their work tasks and is positively linked to improved job satisfaction, performance, and productivity^15^. According to the JD-R model, employees are more likely to engage in their work when they have sufficient resources to meet the demands of their roles effectively. For workers with disabilities, ANTK can serve as a critical job resource, enabling them to overcome challenges and engage more deeply in their work.

Integrating new technologies into the workplace gives workers the tools to mitigate the physical and cognitive demands imposed by their disabilities and limitations. These technologies serve as job resources that help workers overcome barriers and perform their tasks more effectively^16,17^. For workers with visual impairments, adopting new devices – such as screen readers on touchscreen devices, braille displays, and magnification tools- enables them to access information, participate in communication, and manage tasks that would otherwise be inaccessible due to their disability^18^. By enhancing their ability to interact with their work environment, these technologies ultimately support higher levels of work engagement.

Job resources gained from ANTK can help reduce the strain imposed by job demands, enabling workers to complete their tasks more efficiently. Studies have shown that employees with disabilities who have access to appropriate assistive technologies experience less frustration and greater engagement, as they face fewer barriers that hinder their productivity and participation^6^. Workers with visual impairments receive the resources derived from ANTK, which can reduce the cognitive and physical strain associated with their work tasks. Reducing job demands argues that employees are more likely to thrive and remain engaged when not overwhelmed by excessive demands^19^. These innovative technologies also provide a sense of autonomy and control over their tasks, as workers with disabilities gain the ability to perform tasks independently without relying heavily on others for assistance. The increased autonomy that comes with mastering new technologies contributes to work engagement^20^. Workers who have control over how they perform their tasks tend to experience greater satisfaction in their work roles.

Existing literature has extensively explored the positive impact of technology adoption behaviors on workers with visual impairments. When these workers are provided with customized technologies that align with their capabilities, they experience increased efficiency, autonomy, and engagement in their tasks^21^. Armstrong and Murray^22^ found that integrating adaptive technologies tailored to individual needs leads to higher engagement and performance. These findings underscore the importance of aligning technological solutions with the specific requirements of workers with visual impairment to maximize their workplace potential.

Moreover, knowledge acquisition behaviors related to new technologies are crucial for enhancing personal resources, such as self-efficacy and confidence. New technologies equip workers with the knowledge necessary to perform their jobs more effectively. As workers gain expertise in using new technologies, they experience a sense of accomplishment, which motivates them to stay engaged with their work. The development of personal resources facilitates task completion and contributes to higher work engagement by boosting employees’ belief in their ability to achieve success and overcome challenges^23^. By fostering personal growth through ANTK, workers feel more empowered, capable, and involved. Therefore, we posit that ANTK is positively associated with work engagement for workers with visual impairments.

*H1: ANTK is positively associated with work engagement.*

### Mediating effect of person-job fit between ANTK and work engagement

Person-job fit (P-J fit) is the alignment between an individual’s values, skills, abilities, and job demands (Kristof-Brown et al., 2005). A high level of P-J fit occurs when the employee’s capabilities align with the job requirements, thereby increasing job satisfaction, motivation, and engagement. The fit ensures that the job’s demands are compatible with the employee’s abilities, enhancing the likelihood of successfully integrating assistive technologies.

For workers with visual disabilities, successfully adopting new technology and knowledge is closely tied to P-J fit. When assistive technologies align with an individual’s skills and job requirements, they become more effective tools for overcoming barriers and enhancing work performance. This alignment reduces the cognitive and physical strain associated with job demands, fostering a sense of competence and self-efficacy. As a result, workers are more likely to engage deeply in their tasks and experience higher job satisfaction.

While the broader person-environment (P-E) fit framework encompasses multidimensional concepts, such as person-organization or person-group fit, this study focuses specifically on P-J fit, which captures how individuals adapt to challenges in task execution^24^. For this population, the gap between physical limitations and job requirements is the central barrier to engagement. For workers with visual impairments, ANTK acts as a critical enabling resource that bridges the gap between their physical limitations and job requirements. By providing necessary accessibility tools, ANTK actively modifies the demand-ability relationship, creating a state of alignment (P-J fit) that would otherwise be unattainable.

Within the framework, P-J fit remains the most direct mechanism for understanding how ANTK affects work engagement. The effectiveness of new technology in the workplace depends on how well it aligns with the user’s skills and job demands. For example, when employees with visual impairments are provided with updated devices that can be customized to meet their specific needs, it enhances their ability to complete job duties and meet performance expectations^25^. Similarly, Babu and Heath^26^ found that personal assistive mobile technology enhances the capabilities of blind workers to achieve a better task-capabilities fit, which helped organizations better understand the utility of such technology and its alignment with job demands for these workers. Meanwhile, access to updated technology-related knowledge enhances job self-efficacy, which in turn promotes workplace thriving among employees with disabilities^27^. The dual path of technological accommodation and knowledge adoption reduces cognitive strain. It aligns the job demands with workers’ capabilities, making it easier for employees to perform their work effectively and comfortably, thus positively affecting P-J fit.

Key outcomes of P-J fit include job satisfaction, motivation, and work engagement^28^. General employees who feel their competencies align well with their roles report higher job satisfaction. High P-J fit boosts job satisfaction and enhances motivation and engagement. Employees who perceive a strong fit are more committed and involved in their work, leading to increased performance or productivity.

According to the JD-R model, job resources enhance work engagement primarily by shaping employees’ cognitive and motivational states, which serve as proximal mechanisms linking resource acquisition to behavioral outcomes^29^. For workers with visual impairments, ANTK serves as a critical job resource, enhancing access to task information, expanding functional capabilities, and supporting autonomous task execution. These resource gains are internalized through workers’ perceptions of P-J fit, a key psychological appraisal reflecting the extent to which they feel competent and able to meet job demands. When technologies effectively support task completion, workers experience greater alignment between their abilities and job requirements, leading to stronger feelings of efficacy, empowerment, and a more positive attitude toward their work. As JD–R theory suggests, such improvements in perceived capability reduce strain and activate the motivational pathway that sustains engagement.

For these typical workers, ANTK strengthens P–J fit by enabling them to meet job demands, develop new skills, acquire relevant knowledge, and enhance professional competencies. This alignment fosters a sense of accomplishment and reinforces self-efficacy Accordingly, P–J fit represents a theoretically consistent mediating mechanism through which ANTK affects engagement. Therefore, we developed the hypothesis,

*H2: Person-job fit mediates the relationship between ANTK and work engagement.*

### Mediating effect of positive identity as a person with a disability between ANTK and work engagement

People engage in exploring various roles and identities as they strive to form their own sense of self^36,30,31^. Positive identity as a person with a disability refers to the ability to accept one’s disabilities while preserving a constructive self-concept^32^. Existing literature has demonstrated that a positive identity is associated with favorable outcomes for individuals. For instance, findings indicate that individuals who hold a positive sense of self despite their disabilities tend to have fewer symptoms of depression and anxiety compared to their peers^33,34^. Additionally, other studies have shown that a higher level of positive identity can lead to greater confidence in overcoming adversity and forming close relationships with others^41,35,36^.

Positive identity can be viewed as personal resources that contribute to psychological resilience, motivation, and a proactive approach to overcoming challenges^37^. For individuals with visual impairments, a strong and positive sense of their disability identity is particularly important. When they view their disability as an integral part of their self-concept, they are more likely to perceive assistive technologies as empowering tools rather than as crutches or reminders of their limitations^38^. A positive identity helps them navigate the workplace more confidently, reducing the psychological barriers that might otherwise prevent them from fully utilizing new technologies.

When blind workers adopt new technologies and knowledge (ANTK), they often face challenges such as learning new tools, overcoming societal stigma, and adapting their work routines. The belief that their disability is a positive aspect of their identity helps them navigate these challenges, leading to increased work engagement^39^. The relationship between positive identity and work engagement has been verified. Nario-Redmond et al. (2013) found that individuals who developed a positive sense of disability identity were more likely to engage with work tasks and take on new challenges. Similarly, Bogart^28^ showed that workers who accept and embrace their disability report higher well-being and work engagement.

From the standpoint of the social model of disability, disability does not originate from an individual’s impairment but from environmental and societal barriers that restrict participation^40^. This model highlights that disability is shaped by external conditions and societal perceptions rather than merely an individual’s physical or sensory limitation. We propose that ANTK facilitates the development of a positive identity by serving as the mechanism that removes these environmental obstacles. When workers with visual impairments successfully adopt new technologies and knowledge, they experience a tangible reduction in workplace barriers, effectively validating the social model perspective in their daily lives.

Specifically, ANTK empowers individuals to perform tasks independently, reinforcing the belief that their impairment is not a deficit to be fixed, but a difference that can be accommodated^41^. This experience of technological empowerment shifts their self-perception, helping them reject negative societal stereotypes and embrace a positive identity as a person with a disability. In turn, this positive identity—characterized by self-acceptance and a sense of worth—fuels greater work engagement. Workers who view their disability as an integral, positive part of their self-concept feel more competent and motivated to perform their roles effectively. ANTK acts as an antecedent that fosters the positive identity necessary for sustained engagement. Therefore, we hypothesize,

*H3: The positive identity as a person with a disability mediates the relationship between ANTK and work engagement.*

### Serial mediating effect of person-job fit and positive identity as a person with a disability between ANTK and work engagement

The serial mediation model suggests that the relationship between ANTK and work engagement occurs through a chain of effects involving P-J fit and positive identity. Initially, ANTK enhances the P-J fit by providing workers with the tools and knowledge to meet job demands effectively. An enhanced P-J fit strengthens workers’ sense of capability and confidence in their skills, resulting in increased work engagement.

Subsequently, enhanced P-J fit affects positive identity. The social model of disability further supports this sequence by emphasizing that disability identity is shaped by the degree to which environmental barriers are removed. The ‘disability’ experience is often defined by the inability to participate due to environmental mismatch, and we argue that P-J fit serves as a critical ‘mastery experience’ that disrupts this negative feedback loop. When ANTK facilitates a high level of P-J fit, the experience of alignment - where skills successfully meet job demands - validates the worker’s capabilities in a real-world setting. Such behavioral validation effectively challenges internalized stigma, creating the psychological safety necessary for workers to reframe their disability. Instead of viewing their impairment as a deficit that hinders work, the experience of ‘fitting’ the job allows them to integrate their disability into a positive self-concept. Workers with visual impairments perceive their disability as part of their identity that coexists with their professional skills, rather than as a hindrance to workplace success. This positive disability identity, in turn, boosts work engagement by enhancing workers’ self-efficacy, motivation, and autonomy, which are critical factors for sustained engagement.

Furthermore, when workers with disabilities experience high P-J fit and positive identity, they are more likely to approach their work with enthusiasm and commitment. Their ability to perform tasks effectively, combined with their positive self-perception, leads to higher work engagement.

The serial mediation pathway highlights the importance of both external job resources (i.e., ANTK) and internal personal resources (i.e., a positive disability identity) in correlating with engagement. While ANTK offers an effective way to perform work, the extent to which external resources boost engagement depends primarily on person–job fit and the worker’s acceptance of their disability. Based on this proposed serial mediation pathway, we hypothesize,

*H4: There is a serial mediation effect of ANTK on work engagement through person-job fit and the positive identity as a person with a disability.*

## Method

### Sample and procedures

We collaborated with an internet company that develops online games for blind individuals to collect data through questionnaires in China. The company has over 600,000 users with visual impairments. Our sample targeted workers with visual impairments who have worked continuously for over three months. The survey was first created through the SoJump platform, and the company subsequently distributed the survey link to a randomly targeted subset. The company facilitated online data collection through a random sample of the targeted group, using assistive technologies to ensure accessibility. To ensure accessibility for participants, the company conducted an accessibility audit of the survey link before distribution, which includes compatibility testing with commonly used screen-reader software. As a result, the survey could be completed effectively regardless of the severity of visual impairment.

The data were collected using a survey instrument, accompanied by a cover letter that outlined the purpose of the academic research, emphasized voluntary participation, and ensured confidentiality. Participants were asked to complete the surveys independently, fully acknowledging the anonymous and ethical nature of the study. Specifically, the research was approved by the institutional research ethics committee before data collection, and it was conducted in full compliance with the university’s code of research conduct and ethics. The study employed informed consent procedures, provided participants with detailed information sheets, ensured participant anonymity, and securely stored data in accordance with institutional guidelines. In total, 204 valid responses were retained for data analysis after filtering out incomplete or invalid submissions (responses that failed the attention check question were excluded). The demographic profile of the respondents revealed that their average education level was a junior college degree or higher, with an average job tenure of 4.2 years. Among the respondents, 76% were total blindness, and the proportion of respondents with congenital or acquired visual diseases was roughly equal.

### Measures

Each construct was evaluated on a 5-point Likert scale, ranging from 1 (strongly disagree) to 5 (strongly agree). We implemented the back-translation method, ensuring that the original English scales were accurately rendered into Chinese while preserving their intended meaning^42^. This method involves translating the scales into the target language and then back-translating them into the original language to verify the accuracy of the translation. Appendix A shows the measures of all variables.

#### Adopting new technology and knowledge

Adopting new technology and knowledge developed by Bruning and Campion (2018) includes five items. The sample items include “Use new technology or knowledge to enhance communication”. All items capture individuals enacted behaviors toward technology and knowledge. Appendix A shows that this construct demonstrated a high-reliability score (α = 0.920, CR = 0.923).

#### Person-job fit

The person-job fit, as developed by Saks and Ashforth (2002), comprises four items. The sample items include “To what extent do your knowledge, skills, and abilities match the requirements of the job?”. Appendix A shows that this construct demonstrated a high-reliability score (α = 0.870, CR = 0.875).

#### Positive identity as a person with a disability

The positive identity as a person with a disability developed by Bolton and Brookings (1998) includes six items. The sample items include “To what extent do your knowledge, skills, and abilities match the requirements of the job?”. Appendix A shows that this construct demonstrated a high-reliability score (α = 0.805, CR = 0.815).

#### Work engagement

The work engagement developed by Lin^43^ includes six items, including “At my work, I feel full of energy”. Appendix A shows that this construct demonstrated a high-reliability score (α = 0.932, CR = 0.933).

#### Control variables

To address potential alternative explanations for work engagement, we controlled for five demographic variables: gender, education, job tenure, disability severity, and the timing of disability onset.

## Research results

### Confirmatory factor analysis

Table 1 provides the results of confirmatory factor analysis (CFA) and common method bias. The fit indices from confirmatory factor analysis (CFA) through AMOS version 26.0 suggest that a model is well-fitted when the value of χ^2^/df is less than 3, and RMSEA < 0.1, SRMR < 0.08, IFI > 0.90, CFI > 0.90^44^. This study involved the construction of five models. As shown in Table 1, the fitting index for the “four-factor model” (χ2/df = 2.208, RMSEA = 0.083, SRMR = 0.078, IFI = 0.912, CFI = 0.911) shows outstanding results, whereas the other four competing models fail to meet the fundamental criteria.

To assess the presence of common method bias, the study applied the unmeasured single-method factor approach (Podsakoff et al., 2003). Incorporating common method variance (CMV) into the CFA revealed that the fit of the “four-factor model + CMV” did not lead to a significant improvement (SRMR = 0.022 < 0.05, RMSEA = 0.013 < 0.05, CFI = 0.034 < 0.1, TLI = 0.033 < 0.1). Consequently, this data exhibits no significant common method bias.

As shown in Appendix A, the composite reliability (CR) values are 0.923 for ANTK, 0.933 for work engagement, 0.875 for P-J fit, and 0.815 for positive identity. The average variance extracted (AVE) values are 0.707 for ANTK, 0.697 for work engagement, 0.636 for P-J fit, and 0.435 for positive identity. Fornell and Larcker (1981) posited that ideally the CR should be greater than 0.6, and the AVE should be above 0.5, although values between 0.36 and 0.5 are deemed acceptable^46^. Consequently, the CR and AVE satisfy the criteria.

**Table 1:** Results of confirmatory factor analysis.

Model	χ^2^	df	χ2/df	RMSEA	SRMR	IFI	CFI
Single-factor model (ANTK + PJF + PI + WE)	1424.637	189	7.538	0.179	0.136	0.577	0.574
Two-factor model (ANTK + WE; PJF + PI)	1289.971	188	6.862	0.170	0.131	0.623	0.620
Three-factor model a (ANTK + WE; PJF; PI)	1020.849	186	5.488	0.149	0.119	0.715	0.712
Three-factor model b (ANTK; PJF + PI; WE)	712.960	186	3.833	0.118	0.099	0.820	0.818
Four-factor model (ANTK; PJF; PI; WE)	440.729	183	2.208	0.083	0.078	0.912	0.911
Four-factor model + CMV (ANTK; PJF; PI; WE; CMV)	322.957	162	1.994	0.070	0.056	0.945	0.945

ANTK = adopting new technology and knowledge; WE = work engagement; PJF = person-job fit; PI = positive identity as a person with a disability; CMV represents common method variance, and “+” represents factor combination.

### Descriptive analysis and correlations

Table 2 provides a summary of the descriptive analysis and correlation coefficients for ANTK, P-J fit, positive identity, and work engagement. The discriminant validity of each construct is confirmed by the fact that the square root of its AVE surpasses its correlations with other constructs, demonstrating that each construct is adequately differentiated from the others^45^. The mean values of these four variables ranged from 3.378 to 4.133, while the standard deviation varied between 0.842 and 0.899. The findings indicate a positive intercorrelation between ANTK, P-J fit, positive identity, and work engagement.

**Table 2:** Results of descriptive analysis and correlations.

	Mean	SD	1	2	3	4	5	6	7	8	9
1. Gender	1.410	0.493									
2. Education	2.320	1.027	– 0.075								
3. Job tenure	4.200	1.682	– 0.009	0.160*							
4. Disability severity	1.760	0.428	0.027	0.007	0.141*						
5. Disability onset	1.500	0.501	– 0.028	-0.027	0.028	0.109					
6. ANTK	4.133	0.842	– 0.119	0.095	0.118	0.166*	– 0.023	**0.841**			
7. P-J fit	3.642	0.886	– 0.146*	0.099	0.032	– 0.017	0.040	0.358**	**0.797**		
8. Positive identity	3.378	0.899	– 0.022	0.098	– 0.015	– 0.004	0.026	0.396**	0.379**	**0.660**	
9. Work engagement	3.769	0.886	– 0.052	0.037	0.040	0.091	0.148*	0.480**	0.627**	0.476**	**0.835**

*N* = 204. Gender was coded as 1 = male and 2 = female; Education was coded as 1 = junior school degree and below, 2 = high school degree, 3 = junior college degree, and 4 = bachelor’s degree or above; Job tenure was coded as 1 = three to six months, 2 = six months to one year, 3 = one year to three years, 4 = three to five years, 5 = five to ten years, 6 = ten years above; Disability severity was coded as 1 = low vision, 2 = total blindness; Disability onset was coded as 1 = congenital visual disease, 2 = acquired visual disease.

The bold numbers on the diagonal are the square roots of AVE.

*p < 0.05; **p < 0.01.

### Hypotheses test

The results of the linear regression analysis showed that ANTK is positively associated with work engagement (β = 0.494, t = 7.730, *p* < 0.01). Thus, Hypothesis 1 was confirmed. The SPSS PROCESS macro was utilized to implement Model 4 for mediating effect analysis. When P-J fit serves as the mediating variable, ANTK is positively associated with P-J fit (β = 0.371), P-J fit positively affects work engagement (β = 0.527), and ANTK is positively associated with work engagement (β = 0.324). The effect of this indirect path was 0.195 (BootSE = 0.058, BootCI [0.087, 0.310]), indicating that this mediating pathway contributes to 38% of the overall effect, as illustrated in Table 3. Accordingly, the findings provided support for Hypothesis 2.

When positive identity is the mediating variable, ANTK is positively associated with positive identity (β = 0.437). Positive identity, in turn, positively affects work engagement (β = 0.329). Additionally, ANTK positively affects work engagement (β = 0.376). The effect of this indirect path was 0.144 (BootSE = 0.040, BootCI [0.071, 0.225]), indicating that this mediation pathway explains 74% of the overall impact, which supports Hypothesis 3.

**Table 3:** Results of the mediation effect.

Path	Coeff	se	t	*p*	LLCI	ULCI
ANTK →PJF	0.371	0.072	5.159	0.000	0.229	0.512
PJF →WE	0.527	0.056	9.489	0.000	0.418	0.637
ANTK →WE	0.324	0.059	5.460	0.000	0.207	0.441
Total effect	0.519	0.067	7.730	0.000	0.387	0.652
Indirect effect	0.195					
ANTK →PI	0.437	0.072	6.090	0.000	0.295	0.578
PI →WE	0.329	0.063	5.208	0.000	0.204	0.454
ANTK →WE	0.376	0.069	5.478	0.000	0.240	0.511
Total effect	0.519	0.067	7.730	0.000	0.387	0.652
Indirect effect	0.144					

ANTK= adopting new technology and knowledge, PJF= person-job fit, PI= positive identity as a person with a disability, WE= work engagement.

**Fig. 1:**
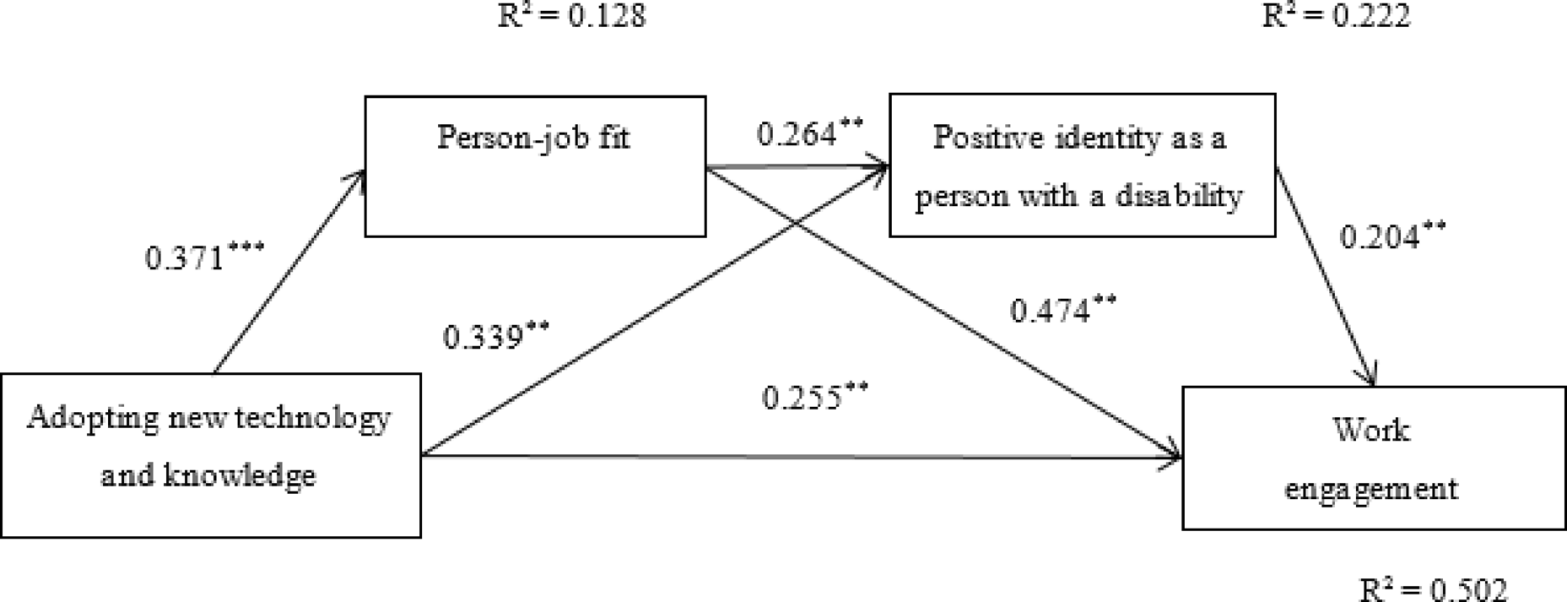
Path analysis of the serial mediation. ***p < 0.001.

Model 6 of the PROCESS macro was applied to investigate the serial mediation effect, and the results are illustrated in Fig. 1. The findings indicate that ANTK is positively associated with person-job fit (b = 0.371, t = 0.072, *p* < 0.001) and positive identity (b = 0.339, t = 0.074, *p* < 0.001). Additionally, P-J fit positively affects positive (b = 0.264, t = 0.069, *p* < 0.001), while ANTK is positively associated with work engagement (b = 0.255, t = 0.061, *p* < 0.001). Furthermore, P-J fit positively affects work engagement (b = 0.474, t = 0.056, *p* < 0.001), and positive identity also has a positive impact on work engagement (b = 0.204, t = 0.056, *p* < 0.001).

The proposed model’s overall effect amounts to 0.519, comprising a direct effect of 0.255 and a total indirect effect of 0.264. As shown in Table 4, three mediating pathways were identified. The first pathway, from ANTK to work engagement through P-J fit, has an effect value of 0.176. The second pathway, where positive identity mediates the relationship between ANTK and work engagement, has an effect value of 0.069. The third pathway, which follows the sequence of ANTK → P-J fit → positive identity → work engagement, has an effect value of 0.020, thereby supporting Hypothesis 4. As shown in Fig. 1, ANTK explains 12.8% of the variance in person-job fit (R² = 0.128). ANTK and person-job fit explain 22.2% of the variance in positive identity (R² = 0.222). ANTK, person-job fit, and positive identity explain 50.2% of the variance in work engagement (R² = 0.502).

**Table 4:** Results of serial mediation.

Path	Effect	Boot standard error	BootLLCI	BootULCI	Mediating effect
Total Indirect Effect	0.264	0.060	0.149	0.383	50.867%
Path 1 Indirect Effect	0.176	0.052	0.081	0.285	33.911%
Path 2 Indirect Effect	0.069	0.025	0.024	0.124	13.295%
Path 3 Indirect Effect	0.020	0.011	0.004	0.045	3.854%

## Discussion

### Theoretical contributions

This study advances the existing theoretical frameworks, particularly the JD-R model, by integrating the adoption of new technology and knowledge as critical job resources for workers with disabilities, specifically those with visual impairments. The JD-R model traditionally explains work engagement as the outcome of balancing job demands and resources. Job resources are elements of the work environment that facilitate goal achievement, mitigate job demands, and support personal growth^29^. Further, the present study demonstrates the applicability of the JD-R model in the underexplored context of workers with visual impairments in disability research. Visual impairment and the adoption behavior of new technologies moderate the salience and effectiveness of job resources in the JD-R model. For workers without disabilities, typical job resources (i.e., autonomy or social support) often operate directly on motivational processes. In contrast, for workers with visual impairments, the motivational potential of these standard resources may be substantially attenuated when core job demands involve inaccessible information or environments. Under such conditions, new technologies and related knowledge serve as basic enabling behavioral resources, restoring accessibility before broader JD-R motivational processes can take effect. Thus, disability status and access to assistive technology may shift the priority of job resources required to foster engagement. This observation warrants explicit testing in future comparative research between workers with and without visual impairments.

This study develops the conceptualization of ANTK by framing it as a multidimensional construct that integrates both material (technology) and cognitive (knowledge) resource acquisition. it represents a proactive strategy through which workers with visual impairments approach opportunities for learning and empowerment while simultaneously reducing barriers to participation. Both material and cognitive aspects are correlated and treated as tightly coupled to behavioral manifestations of the same underlying pattern of action. This dual nature situates ANTK as a critical job resource within the JD–R framework: it mitigates demands and fosters personal growth, autonomy, and identity development. By linking ANTK to both the external environment (technologies, organizational supports) and internal processes (knowledge, competence, self-efficacy), the study enriches the understanding of how technological and knowledge-based resources drive sustainable engagement for workers with disabilities.

By expanding job resources for workers with visual impairments, this study contributes to understanding how ANTK directly affects engagement, autonomy, and job satisfaction among workers with visual disabilities. These findings underscore the crucial role of ANTK in enabling employees to meet the demands of their work, thereby engaging more fully in their roles.

The study also sheds light on P-J fit, which traditionally focuses on aligning employee skills with job demands. The research demonstrates that ANTK can significantly enhance the P-J fit for workers with disabilities, thereby improving their ability to perform their jobs effectively. By demonstrating that the match between workers’ skills and job requirements, facilitated by ANTK, is essential for work engagement, this study underscores the importance of P-J fit in successfully integrating employees with disabilities into the workforce.

Furthermore, the study highlights the role of positive identity as a person with a disability. This positive self-perception enables individuals to engage more fully in their work, suggesting that internal resources, such as self-perception, can significantly impact engagement outcomes. The findings indicate that workers who adopt a social model perspective view disability as an integral part of their identity rather than a deficit to be corrected, and are more likely to experience greater work engagement. This theoretical contribution enriches our understanding of how psychological factors, such as identity, play a crucial role in workers’ interactions with external resources like technology.

### Practical implications

The study provides valuable practical insights for organizations aiming to enhance the work engagement of employees with disabilities. The findings offer actionable insights for managers to support their employees more effectively and foster a more inclusive and productive workplace.

First, organizations should prioritize adopting and integrating assistive technologies to help employees with disabilities perform their tasks effectively. Technologies such as screen readers and magnification software can significantly reduce cognitive and physical strain, making tasks more accessible and manageable^47^. Moreover, organizations should establish training programs to equip employees with the skills necessary to effectively utilize new technologies in their daily work. These programs should focus not only on technological proficiency but also on fostering a growth mindset that encourages employees to see challenges as opportunities for learning and development. Managers should ensure that new technologies are tailored to employees’ needs, enhancing P-J fit and increasing work engagement.

Second, managers need to provide accommodation and adjust roles to ensure that employees with disabilities experience a high level of P-J fit. For example, managers can offer customized or modified workflows that enable workers to use their strengths and abilities effectively. This approach enhances job performance, boosts employee confidence, and motivates them. In practice, managers should also anticipate potential resistance when implementing new technologies by providing accessible training, cultivating a supportive workplace culture, and customizing assistive tools to meet employees’ needs, which can help optimize the benefits of such job resources.

Third, organizations should foster an inclusive culture that supports and celebrates workforce diversity—encouraging employees to view their disability as an integral part of their identity rather than a hindrance can help enhance their self-esteem and self-efficacy^37,48^. Human resource managers can promote positive identity by providing mentorship and career development opportunities to empower these employees. This inclusive environment may encourage employees to engage more deeply in their work, leading to increased job satisfaction and improved performance.

Fourth, beyond providing assistive technologies and supporting the development of positive disability identity, organizations should also consider organizational-level enablers of inclusion. Leadership commitment is particularly critical, as inclusive leaders signal the importance of valuing diversity, shaping organizational culture, and ensuring accountability for disability-related initiatives^49^. Broader culture-building initiatives—such as disability awareness programs, peer mentoring, and inclusive social norms—can enhance belonging and psychological safety for employees with disabilities^50^. Inclusive HR policies can also support retention and workplace integration through strategies such as proactive accessibility audits, tailored training for staff, and partnerships with disabled people’s organizations (DPOs) to co-develop inclusion frameworks^51^. Taken together, these systemic enablers offer a more comprehensive and sustainable approach to workplace inclusion, making the practical recommendations more actionable for managers.

### Limitations and future research directions

The focus of the current study is primarily on workers with visual impairments. While this is an essential and relevant group, future research could expand the sample to include workers with other types of disabilities, such as hearing impairments, cognitive disabilities, or mobility challenges. A broader population would allow a more comprehensive understanding of how ANTK contributes to work engagement across different disability groups.

While the study focused on P-J fit and positive identity as mediators, future research could explore other potential moderators in the relationship between ANTK and work engagement. For example, organizational support or workplace inclusion might also significantly shape how employees with disabilities engage with their work^52–54^. Exploring these additional factors could provide a more nuanced understanding of the mechanisms underlying work engagement for employees with disabilities.

Although this study offers meaningful insights into an underrepresented group, we have to acknowledge that it relies solely on self-reported data and has a limited sample size. Such a cross-sectional design limits the ability to establish causal relationships and may not fully capture the nature of workers’ experiences. Future research could strengthen the evidence by employing longitudinal approaches to capture changes over time, utilizing experimental or quasi-experimental approaches to establish causality, and incorporating multi-source data, such as supervisor and peer ratings, to complement self-reported measures. These approaches would enhance the validity and generalizability of the findings. A further limitation concerns the alternative arrangements of the mediating processes. A more rigorous comparison of competing pathways will require longitudinal or experimental designs capable of establishing temporal precedence. Such work would help determine whether the theorized sequence provides the most robust explanation over time.

Additionally, the research does not explicitly examine the resistance and challenges in technology adoption. Acceptance barriers such as perceived usefulness, personal anxiety, or inadequate organizational support can hinder successful adoption^55,56^. Such challenges resonate with technology acceptance theories, which emphasize that employee perceptions, access to training, and organizational culture are crucial for successful technology integration. Future research could adopt a more nuanced view of ANTK by integrating technology acceptance models into disability and work studies, offering a more comprehensive understanding of the adoption process and its implications for workplace inclusion.

## Conclusion

The findings of this study provide evidence that the adoption of technology and knowledge is associated with higher levels of work engagement among workers with visual impairments. By integrating assistive technologies into their professional environments, these workers can overcome significant workplace barriers, enhancing their ability to perform tasks effectively and independently. However, this research highlights that the benefits of ANTK are not automatic; they depend on how well technology aligns with individual capabilities and work demands. Additionally, the benefits depend on how workers perceive their identity in relation to their disability. Ultimately, this study advances the conversation on digital inclusion and workplace accessibility by demonstrating that ANTK is more than just a technical solution - it is a crucial job resource that, when integrated thoughtfully, fosters engagement, empowerment, and long-term career sustainability for workers with visual impairments.

There is growing societal and governmental recognition of the importance of fostering an inclusive work environment for people with disabilities. The increasing adoption of assistive technologies and workplace accommodations reflects a broader commitment to ensuring that all individuals, regardless of physical limitations, have access to meaningful employment and professional growth. We hope our findings inspire researchers and practitioners dedicated to building truly inclusive workplaces. By integrating thoughtful technology adoption with personalized job design and identity-supportive work environment, organizations and industries can empower workers with disabilities to thrive, contribute, and innovate. A future of total inclusion is possible- and it begins with the collective effort of researchers, practitioners, policymakers, and organizations committed to making a difference.

## Supplementary Information

Below is the link to the electronic supplementary material.

Supplementary Material 1

## Data Availability

Data were provided within the manuscript or supplementary information files. Additional information for data disclosure is accessible upon request from the first author.
