# Supplementary material for: Analyzing the effect of new technology and knowledge adoption on workers with visual impairment through a serial mediation model
Source: Sci Rep. 2026 Jun 29;16:19759. doi: 10.1038/s41598-026-40949-x (PMC13315273; doi:10.1038/s41598-026-40949-x)
Supplement: Supplementary file 1 — Supplementary Material 1 [file 41598_2026_40949_MOESM1_ESM.docx]

Appendix: Table A. Measurements of Variables

| Variables and Items | Factor Loadings | Mean | SD | Cronbach’s Alpha | AVE | CR |
| --- | --- | --- | --- | --- | --- | --- |
| *Adoption of New Technology and Knowledge (Bruning & Campion, 2018)* |  | 4.133 | 0.842 | 0.920 | 0.707 | 0.923 |
| 1. Use new technology or knowledge to enhance communication. | 0.886 |  |  |  |  |  |
| 2. On my own, seek training on new technology. | 0.928 |  |  |  |  |  |
| 3. On my own, seek training to improve my work. | 0.892 |  |  |  |  |  |
| 4. Use new technology or knowledge to automate tasks. | 0.726 |  |  |  |  |  |
| 5. Use new technology or knowledge to structure my work. | 0.752 |  |  |  |  |  |
| *Person-Job Fit (Saks & Ashforth, 2002)* |  | 3.642 | 0.886 | 0.870 | 0.636 | 0.875 |
| 1. To what extent do your knowledge, skills, and abilities match the requirements of the job? | 0.776 |  |  |  |  |  |
| 2. To what extent does the job fulfill your needs? | 0.851 |  |  |  |  |  |
| 3. To what extent is the job a good match for you? | 0.806 |  |  |  |  |  |
| 4. To what extent does the job enable you to do the kind of work you want to do? | 0.753 |  |  |  |  |  |
| *Positive Identity as a Person with a Disability (Bolton & Brookings, 1998)* |  | 3.378 | 0.899 | 0.805 | 0.435 | 0.815 |
| 1. I don't think of myself as a disabled person. | 0.458 |  |  |  |  |  |
| 2. I am proud to be a person with visual impairment. | 0.542 |  |  |  |  |  |
| 3. Being visually disabled has not reduced my enjoyment of life. | 0.715 |  |  |  |  |  |
| 4. My visual impairment is a source of personal strength. | 0.520 |  |  |  |  |  |
| 5. Having visual impairment has not been a problem for me. | 0.836 |  |  |  |  |  |
| 6. I can live a normal life with the visual impairment. | 0.792 |  |  |  |  |  |
| *Work Engagement (Lin, 2010)* |  | 3.769 | 0.886 | 0.932 | 0.697 | 0.933 |
| 1. At my work, I feel full of energy. | 0.878 |  |  |  |  |  |
| 2. In my job, I feel strong and vigorous. | 0.824 |  |  |  |  |  |
| 3. I am enthusiastic about my job. | 0.801 |  |  |  |  |  |
| 4. My job inspires me. | 0.860 |  |  |  |  |  |
| 5. I feel happy when I am working intensely. | 0.807 |  |  |  |  |  |
| 6. I am immersed in my work. | 0.838 |  |  |  |  |  |

Note: AVE = average variance extracted; CR = composite reliability.

References:

Bolton, B., & Brookings, J. (1998). *Development of a Measure of Intrapersonal Empowerment*. *43*(2), 131–142.

Bruning, P. F., & Campion, M. A. (2018). A role-resource approach-avoidance model of job crafting: A multimethod integration and extension of job crafting theory. *Academy of Management Journal*, *61*(2), 499–522. https://doi.org/10.5465/amj.2015.0604

Lin, C.-P. (2010). Modeling Corporate Citizenship, Organizational Trust, and Work Engagement Based on Attachment Theory. *Journal of Business Ethics*, *94*(4), 517–531. https://doi.org/10.1007/s10551-009-0279-6

Saks, A. M., & Ashforth, B. E. (2002). Is job search related to employment quality? It all depends on the fit. *Journal of Applied Psychology*, *87*(4), 646–654. https://doi.org/10.1037/0021-9010.87.4.646
